# Successfully Ablated Atrioventricular Nodal Reentrant Tachycardia in Unconventional Presentation

**DOI:** 10.4103/0975-3583.59985

**Published:** 2010

**Authors:** Wang Jun-Hua, Huang Cong-Chun, Tan Wei-Jie, Liu Chao-Zhong, Sun Jin-Jin, Luo Hui-Lan

**Affiliations:** Department of Cardiology, Air Force General Hospital Beijing 100142, China, PRC

**Keywords:** *Radiofrequency Catheter Ablation (RFCA)*, *Atrioventricular Nodal Reentrant Tachycardia (AVNRT)*, *Atrioventricular Reentrant Tachycardia (AVRT)*

## Abstract

**Background:—:**

Sometime, it’s difficult to distinguish the electrophysiological mechanism of some tachycardia, and so, influencing the efficacy and safety of ablation operation. Therefore, it’s helpful to analysis some tachycardia in particular mechanism, as in this case.

**Methods and results:—:**

A 49 years old Chinese male patient had a history of paroxysmal palpitation for 25 years, and recurred more frequently in the month before admission. Electrocardiogram (ECG) showed no abnormity under sinus rhythm, and showed no specific sign to distinguish its reentrant mechanism when tachycardia running. Electrophysiological examination and the result of successful ablation showed that the retrograde pathway of its reentry was in slow conduction, and from which the reentry started; moreover, after partially ablating, the reentry started from antegrade slow conduction.

**Conclusion:—:**

Careful cardiac electrophysiological examination and paying more attention to inducing conditions of tachycardia are critical to accurately determining the tachycardia mechanism.

A 49 years old Chinese male patient had a history of paroxysmal palpitation for 25 years, and recurred more frequently in the month before admission. Tachycardia usually burst on and off suddenly. The duration of the development was from a few minutes to 10 hours. A history of hypertension was for 4 years. Physical examinations show no abnormal physical signs. Ultrasound examination met with high blood pressure heart changes: mild expansion of the ascending aorta, normal left ventricular systolic function and diastolic dysfunction. Electrocardiogram (ECG) showed no abnormity under sinus rhythm; on paroxysmal tachycardia outburst [Figure 1], narrow QRS complex presenting together with heart rate (HR) 178/ min, interval from QRS wave to P wave (RP interval) was 120ms and RP interval < PR interval (interval from P wave to QRS wave). It’s difficult to distinguish it from atrioventricular reentrant tachycardia.

**Figure 1:**
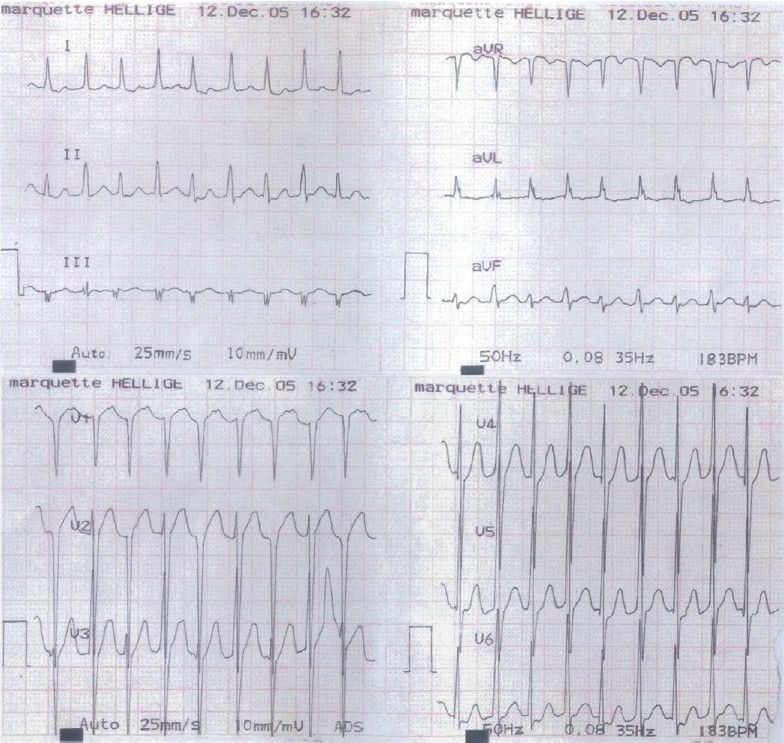
ECG during onset of paroxysmal tachycardia, it’s difficult to classify its mechanism whether is AVRT or AVNRT. Abbreviations: AVRT=atrioventricular reentry tachycardia; AVNRT=atrioventricular nodal reentry tachycardia.

Intracardiac electrophysiological examination: right atrial programmed basic stimulus coupled with cycle length decreasing extrastimulus (S1S2) at 500/280ms induced a narrow QRS complex tachycardia, but it was not accompanied by atrioventricular conduction jump phenomena [Figure 2]. Ventricular wave (V) and atrial wave (A) did not interfuse. VA interval = 120ms. It seems to be a side pathway, but right ventricular grading frequency increment stimulation showed ventricle to atrium decline conduction as well as Wenckebach phenomenon. Tachycardia could not be terminated by ventricular premature beat [Figure 3]. This suggested that there was no atrioventricular side pathway and atrioventricular nodal reentry was very likely. While we tried to improve atrioventricular node by ablating the slow pathway in lower region, atrioventricular junctional rhythm occurred, and the junctional beats decreased with the process of ablation. Subsequent repeated electrophysiological examination showed that under almost the same condition, right atrial programmed S1S2 at 550/270 ms induced atrioventricular conduction jump phenomenon, and the same paroxysmal tachycardia been induced [Figure 4]. We continued to improve atrioventricular node till to the jump phenomenon disappeared. After that not only repeatedly grading frequency increment stimulation but also programmed S1S2 could not induce tachycardia. Then, we repeated these stimulations after intravenous infusion of isoproterenol, still, no tachycardia and atrioventricular conduction jump phenomenon recurred. These electrophysiological examination suggested the success of ablation.

**Figure 2:**
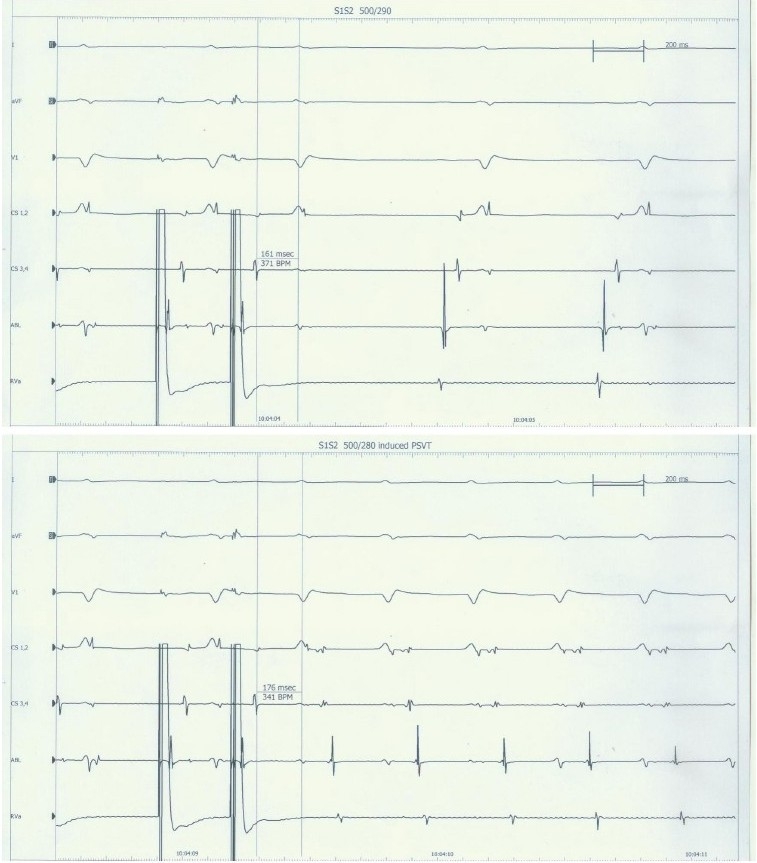
Before ablation, right atrial programmed S1S2 at 500/280 ms induced a narrow QRS complex tachycardia, atrioventricular conduction prolonged only 15ms when S2 decreased from 290 ms to 280 ms, not accompanied by atrioventricular conduction jump phenomenon; and V and A did not interfused during tachycardia. The reentry started from slow retrograde conduction. These properties did not propose conventional AVNRT tachycardia. Abbreviations: PSVT=paroxysmal supraventricular tachycardia; CS1-4=coronary sinus electrograms from distal to proximal; ABL=electrogram recorded by ablating catheter; RVa=electrogram recorded by right ventricle apex catheter; ms (or msec)=millisecond; BPM=beats per second; V=ventricular wave; A=atrial wave; other abbreviations as in Figure 1.

**Figure 3:**
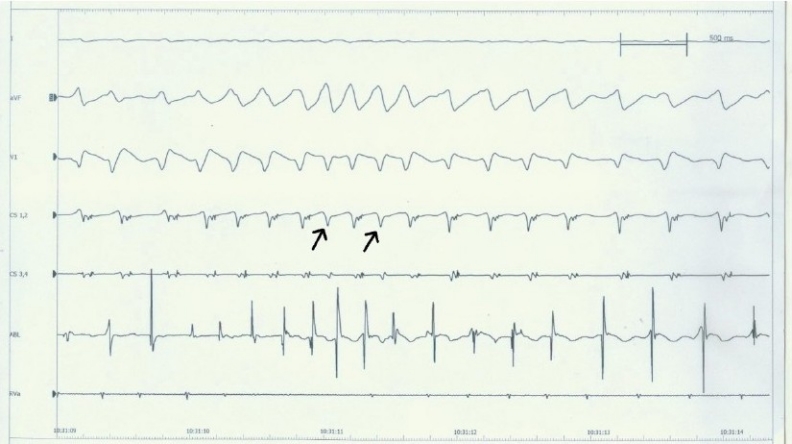
Tachycardia could not be terminated by ventricular premature beat; This did not propose the AVRT mechanism. Abbreviations as in Figure 1 and Figure 2.

**Figure 4:**
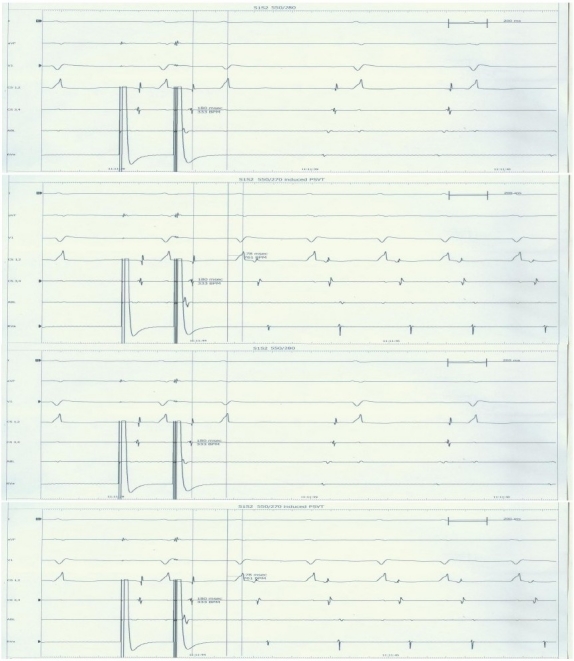
After a certain time of ablation, under almost the same condition of right atrial programmed S1S2 at 550/270 ms as before ablation (500/280 ms), atrioventricular conduction jump phenomenon occurred (78ms prolonged), and the same paroxysmal tachycardia induced. This suggested that the reentry started from antegrade slow conduction.

## DISCUSSION

According to the electrophysiological examination together with the successful ablation results, we concluded that the mechanism for tachycardia of this patient is atrioventricular nodal reentry.^1^–^2^ Different from conventional atrioventricular node dual pathways, for this patient, the retrograde conduction of the atrioventricular node was slow. This situation is relatively rare. The reason why right atrial programmed S1S2 could induce tachycardia but not accompanied with atrioventricular conduction jump phenomenon, is that the reentry originated from slow ventricular-atrial retrograde conduction, rather than from slow atrioventricular antegrade conduction. After partially improved, the electrophysiological characteristic of conduction pathways of reentrant tachycardia had altered, accordingly, the reentry of paroxysmal tachycardia stemmed from slow anterograde conduction, so presented conventional atrioventricular conduction jump phenomenon.

Tips of the case: The complexity of some rare types of supraventricular tachycardia increases a certain difficulty to ablation procedure.^3^–^4^ A careful analysis of ECG, and careful cardiac electrophysiological examination and paying more attention to inducing conditions of tachycardia are critical to accurately determining the tachycardia mechanism.
